# New Enhancing MRI Lesions Associate with IL-17, Neutrophil Degranulation and Integrin Microparticles: Multi-Omics Combined with Frequent MRI in Multiple Sclerosis

**DOI:** 10.3390/biomedicines11123170

**Published:** 2023-11-28

**Authors:** Zsolt Illes, Malene Møller Jørgensen, Rikke Bæk, Lisa-Marie Bente, Jørgen T. Lauridsen, Kirsten H. Hyrlov, Christopher Aboo, Jan Baumbach, Tim Kacprowski, Francois Cotton, Charles R. G. Guttmann, Allan Stensballe

**Affiliations:** 1 Department of Neurology, Odense University Hospital, 5000 Odense, Denmark; 2 Department of Clinical Medicine, University of Southern Denmark, 5230 Odense, Denmark; 3 Institute of Molecular Medicine, University of Southern Denmark, 5230 Odense, Denmark; 4 Brain Research—Inter Disciplinary Guided Excellence (BRIDGE), University of Southern Denmark, 5230 Odense, Denmark; 5 Department of Clinical Immunology, Aalborg University Hospital, 9220 Aalborg, Denmark; maljoe@rn.dk (M.M.J.); rikke.baek@rn.dk (R.B.); 6 Division Data Science in Biomedicine, Peter L. Reichertz Institute for Medical Informatics of TU Braunschweig and Hannover Medical School, 38106 Braunschweig, Germany; lisa-marie.bente@plri.de (L.-M.B.); tim.kacprowski@plri.de (T.K.); 7 Braunschweig Integrated Centre for Systems Biology (BRICS), TU Braunschweig, 38106 Braunschweig, Germany; 8 Department of Business and Economics, University of Southern Denmark, 5230 Odense, Denmark; jtl@sam.sdu.dk; 9 Department of Health Science and Technology, Aalborg University, 9220 Aalborg, Denmark; cabo@hst.aau.dk; 10 Sino-Danish Center for Education and Research, University of Chinese Academy of Sciences, 101408 Beijing, China; 11 Department of Mathematics and Computer Science, University of Southern Denmark, 5230 Odense, Denmark; jan.baumbach@uni-hamburg.de; 12 Institute for Computational Systems Biology, University of Hamburg, 20148 Hamburg, Germany; 13 Service de Radiologie, Centre Hospitalier Lyon-Sud, France/CREATIS, Université de Lyon, 69007 Lyon, France; francois.cotton@chu-lyon.fr; 14 Center for Neurological Imaging, Brigham and Women’s Hospital, Boston, MA 02115, USA; guttmann@bwh.harvard.edu; 15 Clinical Cancer Center, Aalborg University Hospital, 9220 Aalborg, Denmark

**Keywords:** biomarker, MRI, mass spectrometry, EV array, endothelial stress, blood brain barrier, IL-17, IL-1β, multiple sclerosis, coagulation, complement, gadolinium, enhancing lesion, plasma

## Abstract

Background: Blood–barrier (BBB) breakdown and active inflammation are hallmarks of relapsing multiple sclerosis (RMS), but the molecular events contributing to the development of new lesions are not well explored. Leaky endothelial junctions are associated with increased production of endothelial-derived extracellular microvesicles (EVs) and result in the entry of circulating immune cells into the brain. MRI with intravenous gadolinium (Gd) can visualize acute blood–barrier disruption as the initial event of the evolution of new lesions. Methods: Here, weekly MRI with Gd was combined with proteomics, multiplex immunoassay, and endothelial stress-optimized EV array to identify early markers related to BBB disruption. Five patients with RMS with no disease-modifying treatment were monitored weekly using high-resolution 3T MRI scanning with intravenous gadolinium (Gd) for 8 weeks. Patients were then divided into three groups (low, medium, or high MRI activity) defined by the number of new, total, and maximally enhancing Gd-enhancing lesions and the number of new FLAIR lesions. Plasma samples taken at each MRI were analyzed for protein biomarkers of inflammation by quantitative proteomics, and cytokines using multiplex immunoassays. EVs were characterized with an optimized endothelial stress EV array based on exosome surface protein markers for the detection of soluble secreted EVs. Results: Proteomics analysis of plasma yielded quantitative information on 208 proteins at each patient time point (*n* = 40). We observed the highest number of unique dysregulated proteins (DEPs) and the highest functional enrichment in the low vs. high MRI activity comparison. Complement activation and complement/coagulation cascade were also strongly overrepresented in the low vs. high MRI activity comparison. Activation of the alternative complement pathway, pathways of blood coagulation, extracellular matrix organization, and the regulation of TLR and IGF transport were unique for the low vs. high MRI activity comparison as well, with these pathways being overrepresented in the patient with high MRI activity. Principal component analysis indicated the individuality of plasma profiles in patients. IL-17 was upregulated at all time points during 8 weeks in patients with high vs. low MRI activity. Hierarchical clustering of soluble markers in the plasma indicated that all four MRI outcomes clustered together with IL-17, IL-12p70, and IL-1β. MRI outcomes also showed clustering with EV markers CD62E/P, MIC A/B, ICAM-1, and CD42A. The combined cluster of these cytokines, EV markers, and MRI outcomes clustered also with IL-12p40 and IL-7. All four MRI outcomes correlated positively with levels of IL-17 (*p* < 0.001, respectively), and EV-ICAM-1 (*p* < 0.0003, respectively). IL-1β levels positively correlated with the number of new Gd-enhancing lesions (*p* < 0.01), new FLAIR lesions (*p* < 0.001), and total number of Gd-enhancing lesions (*p* < 0.05). IL-6 levels positively correlated with the number of new FLAIR lesions (*p* < 0.05). Random Forests and linear mixed models identified IL-17, CCL17/TARC, CCL3/MIP-1α, and TNF-α as composite biomarkers predicting new lesion evolution. Conclusions: Combination of serial frequent MRI with proteome, neuroinflammation markers, and protein array data of EVs enabled assessment of temporal changes in inflammation and endothelial dysfunction in RMS related to the evolution of new and enhancing lesions. Particularly, the Th17 pathway and IL-1β clustered and correlated with new lesions and Gd enhancement, indicating their importance in BBB disruption and initiating acute brain inflammation in MS. In addition to the Th17 pathway, abundant protein changes between MRI activity groups suggested the role of EVs and the coagulation system along with innate immune responses including acute phase proteins, complement components, and neutrophil degranulation.

## 1. Introduction

Endothelial dysfunction is defined as a stressed endothelium with a pro-inflammatory phenotype and can be characterized by biomarkers derived from different molecular pathways. In multiple sclerosis (MS), minor endothelial lesions and blood–barrier (BBB) breakdown are common phenomena due to inflammation. Brain magnetic resonance imaging (MRI) with intravenous gadolinium (Gd) may show contrast enhancement as a consequence of BBB breakdown, and pinpoint areas of active inflammation in MS lesions [1,2].

Endothelial cells compose the most luminal boundary of BBB and are essential in maintaining its integrity. Endothelial cells are also major players in cellular trafficking thus controlling central nervous system (CNS) inflammation. Endothelial cells are one of the major producers of nitric oxide (NO) through the activation of inducible NO synthase (iNOS) [3]. The role of endothelial cells has not been fully explored in patients with MS, although they may provide a tool to test the status of the BBB and disease by the examination of endothelial stress, activation, and function. During inflammation, endothelial cells are thought to facilitate the transport of activated leukocytes, and the disease-related stress condition causes leaky endothelial junctions; it initiates an increased expression of circulating adhesion molecules on the endothelial cell membrane and increased production of endothelial microvesicles (EV) [3,4]. In inflammatory diseases of the CNS, the EVs are present in the cerebrospinal fluid (CSF), and in whole blood fractions such as plasma and serum and are commonly thought to be potential “biomarker treasure chests” [5,6].

Exploring EVs derived from endothelial cells provides a promising approach to examining endothelial cell function and dysfunction. Important in intercellular communication, EVs and the subset hereof exosomes are differentially released by cells in stressed environments [7]. EVs are fragments of cellular membranes shed from stressed or damaged endothelial cells; they reflect endothelial inflammation and correlate with the functional capacity of the endothelium [8,9]. EVs can promote monocyte adhesion and transendothelial migration [10]. The number of EV-monocyte complexes is increased in relapsing MS compared with remission; such EV-monocyte complexes yielded a higher transendothelial migration rate compared with monocytes of control subjects, thus suggesting that EVs could be an important element in driving MS activity as well as a disease marker [11]. The antigen-based protein array (EV array) can detect proteome changes within EVs derived from stressed endothelial cells as well as known inflammation and cellular markers [12]. This antigen array identifies several EVs based on different combinations of “stress” surface proteins as well as CD markers, thus it provides a sensitive tool to quantify “stressed” endothelial EVs and detect endothelial stress. This antigen array is combined with biomarkers reflecting NO metabolism and endothelial activation to create an “endothelial stress package”.

The severity of MS-related CNS damage as assessed clinically and by MRI is an important factor reflecting quantitative and qualitative aspects of functional reorganization, interacting with age at disease onset, disease duration, and disease-modifying therapies [13,14]. Inflammation around post-capillary venules and related BBB disruption is exploited by MRI examination in MS, where enhancement on post-contrast T1-weighted scans suggests active inflammation due to leakage of the intravenously administered contrast material into the parenchyma through the damaged BBB [6]. The recent 2017 McDonald diagnostic criteria include MRI to establish dissemination in time (DIT) and/or dissemination in space (DIS) [1]. The recommended diagnostic workup based on the 2021 MAGNIMS criteria suggests a multi-sequence MRI protocol preferably at 1.5 or 3T, including T2-w and/or FLAIR sequence, along with a T1-w sequence after the administration of a paramagnetic contrast-enhancing agent, typically gadolinium (Gd). Lesions demonstrate signal enhancement in the event of disruption of the BBB. In addition to diagnosis, MRI is used to monitor patients to evaluate treatment efficacy, and in situations of unexpected clinical events or potential adverse events [15,16]. Moreover, MRI provides potential prognostic markers and can be used to derive outcome measures for evaluating the prognostic potentials of soluble biomarkers in the long term [17,18]. While Gd-enhancing lesions are an important representation of acute inflammatory activity, T2/FLAIR lesions have less specific pathology and may reflect several factors including inflammation, demyelination, edema, gliosis, and axonal loss [19]. Nevertheless, the formation of new T2/FLAIR lesions appears to be always associated with Gd-enhancement on post-contrast T1-w images [1]. This is caused by the increased permeability of the BBB. Indeed, Gd-enhancement of newly formed lesions correlates with the migration of immune cells and the formation of an acute lesion [19,20]. Serial monitoring has shown that about 80% of the newly formed T2/FLAIR hyperintense signals disappear after 10 weeks, while Gd-enhancement usually resolves within a month [21,22,23].

The number of Gd-enhancing lesions is predictive of long-term outcomes, but the sensitivity of post-contrast images in detecting ongoing inflammation is limited (especially when MRI is performed infrequently, such as in clinical practice) and depends even on the dose of the injected contrast material. Therefore, additional biomarkers detecting blood–barrier damage may enable frequent and sensitive monitoring of disease activity in MS.

By using a multiarray technology of soluble biomarker detection, EV array, and quantitative proteomics, we have examined molecular markers of inflammation, BBB disruption, endothelial stress, and cell migration in the plasma of patients, who underwent weekly multimodal MRI for 8 weeks [1]. We studied associations between the levels of soluble molecular markers and markers of MRI activity to examine systemic changes related to BBB disruption and endothelial dysfunction.

## 2. Materials and Methods

### 2.1. Collection of Sample Material (MRI, Plasma)

Five patients were enrolled in this study (four women and one man; mean age: 38.2 years old; mean disease duration: 6.6 years; mean Expanded Disability Status Scale (EDSS): 2) with definite RMS6 between March 2009–September 2010 (Neurological Hospital, Hospices Civils de Lyon, France). No disease-modifying treatment was used. Enrollment, blood sample collection, and MRI acquisition were published earlier [1].

Blood samples were obtained from the cubital vein before each scan. Plasma samples were collected without signs of hemolysis and stored in aliquots at −80 °C prior to processing.

The longitudinal prospective study (ClinicalTrials.gov Identifier: NCT00861172) was approved by an ethics committee (CPP Lyon Sud-Est IV) and the French Health Products Safety Agency (AFSSAPS) [1]. All patients signed an informed consent form approved by the ethics committee (Institutional Review Board).

### 2.2. MRI Analysis of Five Patients

Frequent high-resolution MRI consisted of a baseline (BL) MRI followed by seven consecutive weekly MRIs (BL to day 49) on five RRMS patients as previously published [1]. MRI acquisitions were performed on a 3T MRI system (Achieva 3T, Philips Medical Systems, Amsterdam, The Netherlands). A standard dose of 0.1 mmol/kg Gadobutrol (Gadovist) was administered during each MRI session 60 s before the 3D T1 acquisition and the 3D FLAIR and 3D-T1-Gd images used for this study. Analysis of MRI data included: the total number of Gd-enhancing lesions, the number of new Gd-enhancing lesions, the number of Gd-enhancing lesions with maximal Gd intensity at each time point, and the number of new FLAIR lesions. The patients were post-analysis divided based on the level of MRI activity measurement (low: no Gd-enhancing lesions; medium: 8–26 Gd-enhancing lesions; high: 116 Gd-enhancing lesions) across 49 days.

### 2.3. Proteomics—Sample Preparation and UPLC-Tandem MassSpec Analysis

A modified FASP protein digestion for plasma with trypsin was used, with phase inversion surfactant removal according to Nguyen et al. [24]. For each biological replicate sample, a total of 100 µg protein was transferred to individual YM-10 kDa spin filters (Millipore, Billerica, MA, USA) and buffer was exchanged to 5% SDC in 50 mM triethylammonium bicarbonate (TEAB) by centrifugation. All centrifugation steps were performed at 14,000× *g* for 15 min at 4 °C. The proteins were then subjected to alkylation with 12 mM tris(2-carboxyethyl)phosphine (Thermo Scientific, Waltham, MA, USA) for 30 min at 37 °C, and reduction with 50 mM chloroacetamide (Sigma-Aldrich, St. Louis, MO, USA) for 20 min at 37 °C in the dark. The reducing and alkylating agents were dissolved in 120 mM SDC in 50 mM TEAB, pH 8.5, and centrifuged after each step. In preparation for digestion, 100 µL digestion buffer (0.5% in 50 mM TEAB) was added to the spin filter and centrifuged. A 1:50 (*w*/*w*) trypsin:protein ratio dissolved in 50 µL digestion buffer was added to the spin filter, and the samples were digested overnight at 37 °C. The flow-through containing the tryptic peptides was recovered by centrifugation followed by a phase separation performed with 3:1 (*v*/*v*) ethyl acetate:sample, acidified by addition of formic acid (FA) to a final concentration of 0.5%. Total phase separation was achieved by 1 min vortexing followed by centrifugation. The collected aqueous phase was vacuum centrifuged overnight and stored at −80 °C until time of analysis.

### 2.4. Proteomics—Mass Spectrometry Analysis

The loaded sample amounts were normalized using A280 on a NanoDrop 1000 (Thermo Scientific, Wilmington, DE, USA), and 5 µg total peptide material was analyzed per UPLC-MassSpec analysis.

The samples were analyzed using a UPLC-nanoESI HCD MassSpec/MassSpec setup with an RSLC nanopump module. The system was coupled online with an emitter for nanospray ionization (new objective picotip 360-20-10) to a QExactive Plus mass spectrometer (Thermo Scientific, Waltham, USA). The peptide material was loaded onto a 2 cm trapping reversed phase Acclaim PepMap RSLC C18 column (Dionex, Sunnyvale, CA, USA) and separated using an analytical 50 cm reversed phase Acclaim PepMap RSLC C18 column (Dionex). Both columns were kept at 40 °C. The sample was eluted with a gradient of 96% solvent A (0.1% FA) and 4% solvent B (0.1% FA in ACN), which was increased to 8% solvent B on a 5 min ramp gradient and subsequently to 30% solvent B in 35 min ramp gradient, at a constant flow rate of 300 nL/min. The mass spectrometer was operated in positive mode (*m*/*z* 375–1400), selecting up to 12 precursor ions with a mass window of m/z 1.6 based on highest intensity for HCD fragmenting, at a normalized collision energy of 27. Selected precursors were dynamically excluded for fragmentation for 30 s.

### 2.5. Proteomics—Data Analysis

A label-free quantification (LFQ) analysis of the plasma was performed in MaxQuant 1.5.7.0 by searching the data files against the Uniprot *Homo sapiens* reference proteome with isoforms (UP000005640, protein count 70,952). All standard settings were employed with carbamidomethyl (C) as a static modification and protein N-terminal acetylation, deamidation (NQ), and Oxidation (M) were included as variable modifications. All reported proteins were identified with an <1% FDR, to ensure only high-confidence protein identifications. Result files from MaxQuant were analyzed in Perseus v1.6.12.0 where reverse hits were removed from further analysis, and the data were log2-transformed. Furthermore, more than two unique peptides were required for valid protein identification and quantification to ensure high-quality data. To conduct a principal component analysis (PCA), missing values (i.e., proteins where a quantification value was not obtained for a given replicate analysis) were imputed with values from a normal distribution (width 0.3 and downshift 1.8) to simulate signals from low abundant proteins. All raw data and unfiltered search data have been made public through the PRIDE ProteomeXchange consortium with the dataset identifier PXD035422 [25].

### 2.6. EV Array Analysis—Preparation of EV Stress Optimized EV Array

Assembly of an optimized EV array was performed by reviewing the literature as well as quantitative proteome analysis of hypoxic (5%/21% O_2_) stressed HBMEC cells [26]. Production of microarrays: Microarray printing was performed on a SpotBot^®^ Extreme Protein Edition Microarray Printer (Sunnyvale, ArrayIt, CA, USA) as previously described [27].

Antibodies for phenotyping of vesicles: For the phenotyping, a total of 33 anti-human antibodies were used. They are listed in the following with the corresponding product number (#) or clone. From R&D Systems (Minneapolis, MN, USA): CD82 (#423524) and TNFRI (#DY225). From Bio-legend: CD63 (MEM-259) and HLA-DR (L243). From LifeSpan BioSciences, Inc. (Seattle, WA, USA): CD9 (#LS-C35418) and CD81 (#LS-B7347). From Abcam (Cambridge, MA, USA): Flotil-in-1 (#Ab41927). From Haematologic Technologies, Inc. (Essex Junction, VT, USA): Lactadherin (#BLAC-1200). From BD Biosciences: CD3 (Hit3a). From Abbiotec (San Diego, CA, USA): CD11a (HI111). From eBioscience: ICAM-1 (R6.5). All antibodies for the phenotyping were printed in triplicates at 87.5–200 µg/mL diluted in PBS containing 5% glycerol.

Antibodies for semi-quantification of vesicles: For the semi-quantification, only anti-CD9, anti-CD63, and anti-CD81 were printed on the microarray slides [27]. Catching and visualization: The entire procedure was performed as described previously [28]. In short, the printed slides were blocked and incubated with the EV-containing sample, followed by detection of bound EVs with biotinylated anti-CD9, -CD63, and -CD81, and Cy5-labelled streptavidin.

### 2.7. EV Array Analysis—Interpretation and Statistical Analysis of Data

Data analysis: Creation of graphs and statistical calculations were carried out using GraphPad Prism (version 6.04, GraphPad Software, Inc., San Diego, CA, USA), SigmaPlot (version 11, Systat Software Inc., San Jose, CA, USA), and Excel (version 2013, Microsoft, Redmond, WA, USA). Heatmaps were produced using Genesis (version 1.7.6, IGB TU Graz, Graz, Austria). For a given antibody spot, the signal intensity was calculated as the mean signal of triplicate spots in relation to the sample signal of the negative spot (PBS) in triplicate. For each spot, the signal intensity was calculated by subtracting the mean of the background (no sample/blank, washing buffer) from the mean of the foreground (spot signal). Before visualization and calculation of linearity, the antibody signal intensities were converted to log space by log2 transformation. The EV “retention” percentage, used with the semi-quantitative data, was calculated as: (Signal in EV starting compartment/total signal in upper and lower compartment) × 100.

### 2.8. Multiplex Analysis for Neuroinflammatory Profiling

Cytokine and chemokine concentrations were determined by multiplex technology of plasma samples (25 μL/sample), parallel to those used for proteomics and EV array, using the MSD human V-PLEX Neuroinflammation Panel 1 Kit (Mesoscale Discovery, Rockville, MD, USA) and a SECTOR Imager 6000 (Mesoscale Discovery) Plate Reader according to the manufacturer’s instructions (PIGF, Tie2, VEGF, VEGF-C, VEGF-D, bFGF, sFlt-1, SAA, ICAM-1, VCAM-1, CCL11/eotaxin, CCL26/eotaxin-3, CXCL10/IP-10, CCL2/MCP-1, CCL13/MCP-4, CCL22/MDC, CCL3/MIP-1α, CCL4/MIP-1β, CCL17/TARC, GM-3CSF, IL-12/IL-23p40, IL-12p70, IL-15, IL-16, IL-17A, IL-1α, IL-1β, IL-5, IL-7, TNF-β, TNF-α, IFN-γ, IL-10, IFN-γ, IL-10, IL-13, IL-2, IL-4, IL-6, IL-8). Samples were diluted two-fold in manufacturer-supplied Diluent 41 prior to measurement. Data were analyzed using MSD Discovery Workbench software (v4.0).

### 2.9. Statistical Analysis

A PCA with two components was made to visualize the major source of variation in the plasma protein expression, and volcano plots were made to show log2 fold change in plasma proteins between cytokines and proteome profiles, respectively. Benjamini–Hochberg adjusted *p*-values smaller than 0.05 were considered significant. Heatmaps with hierarchical clustering were also made in Perseus [29].

A sparse partial least squares discriminant analysis (sPLS-DA) was adapted from the MixOmics R package to identify what proteins could discriminate between the three groups of MRI activity (low, medium, high) according to multiplex and proteomics grouping [30]. The performance of an initial sPLS-DA model with 5 components was assessed using the perf function with 3-fold cross-validation and 50 repeats to identify the optimal number of components. The optimal number of variables on each component was then determined using the tune.splsda function with 3-fold cross-validation and 500 repeated. The number of variables tested was 5 to 25 in increments of 5. The loadings variables that were selected on each component were then visualized using the plotloadings function, and a heatmap of the selected variables was made using the cim function with hierarchical clustering based on Euclidean distance and complete linkage.

Pearson correlation was used to examine correlation among soluble biomarkers, EV markers, and markers of MRI activity. To identify the most relevant combination of biomarkers for the prediction of MRI findings, we calculated AUC-weighted feature importance in a cross-validated Random Forest. The performance of the created models was evaluated using the area under the receiver–operating characteristic curve (AUROC). A linear mixed model was implemented and evaluated to predict the number of newly occurring lesions of each type (the number of new FLAIR lesions, the number of new Gd-enhancing lesions, the total number of Gd-enhancing lesions, and the number of Gd-enhancing lesions with max Gd intensity) while taking effects of different patients and timepoints into account.

## 3. Results

**Figure 1:**
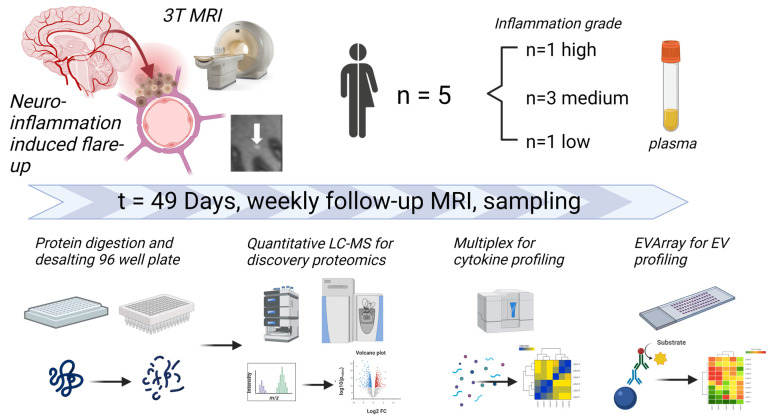
Experimental workflow. Forty plasma samples (repeated measures discovery cohort) from five untreated MS patients with different levels of MRI activity based on the number of Gd-enhancing lesions were prepared for discovery proteomics profiling by quantitative mass spectrometry, multiplex cytokine profiling, and EV array-based liquid biomarker analysis.

In this work, we systematically investigated inflammatory changes in the plasma proteome of five untreated MS patients undergoing frequent high-resolution MRI with Gd: we used mass spectrometry-based quantitative plasma profiling, multiplex-based cytokine and chemokine profiling, and soluble exosome marker assay optimized for oxidative stress-related biomarkers (Figure 1; Supplementary Tables).

### 3.1. The Inflammatory Profile in Patients Reflects the Number of New Gd-Enhancing and FLAIR Lesions

**Figure 2:**
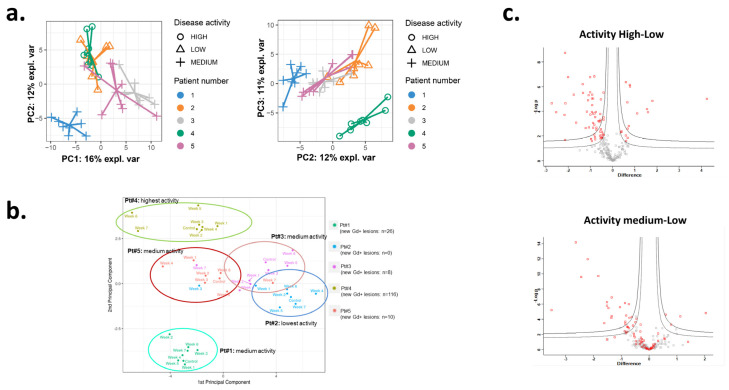
Plasma marker expression clusters based on different MRI activity. (**a**) Principal component visualization of the principal components 1-2 and 2-3 of proteomics expression levels in the plasma. (**b**) Principal component visualization of the first two principal components of biomarker expression levels in the plasma determined by Mesoscale in 5 patients (Pt1-5) with different MRI activity based on the number of gadolinium-enhancing lesions. Each point corresponds to a patient examination at the indicated time point (control = baseline and weekly samples). Colors correspond to the five examined patients. Control indicates baseline. (**c**) Quantitative plasma proteome comparison of inflammation levels (-log10, *p* > 0.05 red dots; log2 fold change; gray dots unchanged).

The discovery cohort containing plasma samples from baseline (BL) to week 7 (day 49) was analyzed by quantitative proteomics without depletion of the most abundant proteins to preserve the full integrity of the plasma proteome. We initially investigated the individual proteomic profiles at each time point by PCA where each patient’s samples cluster together, suggesting that the major source of variation in the plasma proteome was interindividual variation and disease activity rather than the different time points (Figure 2a).

Similarly, using PCA we examined if plasma chemokine–cytokine biomarker levels could separate patients based on MRI activity [31]. The inflammation/endothelial stress/migration-associated plasma chemokine-cytokine levels likewise separated the patients with high, medium, and low numbers of new Gd-enhancing lesions over 8 weeks (Figure 2b). The chemokine–cytokine profiles also indicated the individual profile of biomarkers as they clustered the individual patients. Nevertheless, levels of soluble markers in two patients with medium MRI activity overlapped, and some overlap was also seen with the patients with low MRI activity.

Thus, we grouped the individual proteomic samples according to associated MRI activity (low, medium, high). We observed an overall high LFQ Pearson correlation between 0.89–0.99 and the identification of 340 protein groups at 1% FDR. We were able to quantify a total of 204 proteins after strict filtration and 26–71 proteins were differentially regulated (DEP) as over and underabundant between the three activity levels across all time points (Figure 2c Hawaii plot; Supplementary Tables). Investigating the same DEPs, many of the proteins were overlapping in comparisons between each comparison of MRI activity “low vs. high” and “low vs. medium”.

**Figure 3:**
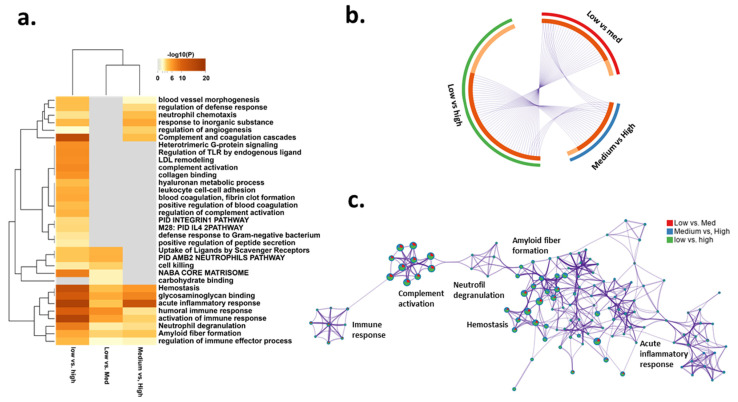
Proteome-based functional enrichment analysis and profiling of MS patients. (**a**) Functional enrichment analysis of regulated proteins in plasma with corresponding functional terms (the heatmap cells are colored by their *p*-values); (**a**) Circos plot indicating how DEPs from each comparison group overlap. On the outside, each arc represents the identity of each protein list of DEPs, and the inside the overlap of shared and unique DEPs. Dark orange color represents the DEPs that are shared by multiple lists, and light orange color represents unique DEPs. (**c**) Enrichment network analysis based on regulated protein lists. Expression levels of soluble plasma proteomes at different time points with low, medium, and high levels of MRI activity and observed expression indicated at protein level and functional grouping level.

Next, we wanted to address the biological function of the DEPs from the comparisons of the three MRI activity groups. The low-to-medium MRI activity consisted of 35 DEPs, the low-to-high MRI activity of 70 DEPs, and the medium-to-high MRI activity of 26 DEPs, respectively. We investigated DEPs by functional enrichment analysis to identify key biological processes that are unique or shared by the three MRI activity comparison groups. We observed the highest enrichment in the low vs. high and medium vs. high comparisons indicating the uniqueness of the high-activity MRI group again (Figure 3a). Complement activation and complement/coagulation cascade were strongly represented in the low vs. high comparison. Activation of the alternative complement pathway, pathways of blood coagulation, extracellular matrix organization, and regulation of TLR and IGF transport were unique for the low vs. high comparison. The expressed DEPs highly overlapped between each group on the expressed protein level as well as functional group level (Figure 3b) indicative of shared biological activity changes between each patient subset. However, the highest number of unique DEPs was observed in the low vs. high MRI activity comparison, while more DEPs overlapped between the low vs. medium and the medium vs. high comparisons (Figure 3b). Investigating the individual DEPs, although several pathways overlapped, such as “inflammatory response” (A2M, AHSG, F12, FN1, HP, HPR, IGHG1, LBP, ORM2, SERPINF2, S100A8, SAA1, SAA2, CD5L, CD14, CD44, ECM1, LYZ, PF4, PRDX2), “complement activation” (C7, C9, CFH, IGHA1, IGHA2, IGHG1, IGHG2, IGHG3, IGHG4, IGHM, IGLC1, CFP, FCN3, IGHV5-51, IGHV4-61, IGHV3-49, IGHV3-23, IGHV3-20, IGHV2-26, C1RL, HRG, JCHAIN, LYZ,PF4, DBH, LBP, PRDX2, GPLD1, HP,S100A8, DCD, AZGP1, APOB, CD14, LRG1, APOA4, F12, FETUB, PIGR, AHSG, CD5L, PRG4, IGLV2-18, IGKV1-8, FN1, CETP, “neutrophil degranulation” (AHSG, CD14, CD44, HP, LYZ, ORM2, CFP, PIGR, QSOX1, S100A8, TTR, LRG1), “platelet activation” (FN1, HRG, PF4, SAA1, VWF, SERPIND1, LBP, ORM2, PRDX2, CD14, LUM, SERPINF2, DBH, S100A8) and “amyloid fiber formation” (APOA4, LYZ, SAA1, TGFBI, TTR), they were most strongly represented in the low vs. high comparison (Figure 3a). When further investigating the known functional networks and presence in each DEP comparison group, a high degree of overlap was observed, although with the dominance of the low vs. high MRI activity patients (Figure 3c).

**Figure 4:**
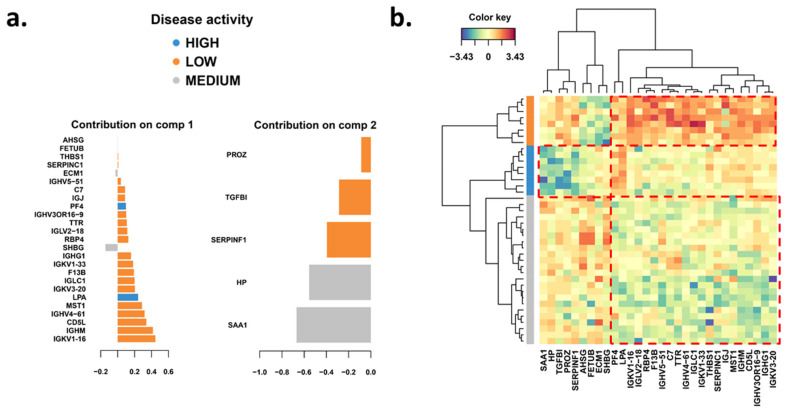
Statistical analysis of DEPs to identify MRI activity groups. (**a**) Partial Least-Squares Discriminant Analysis and proteins associated with components one and two. Clusters indicated according to dendrograms (**b**) Clustered heatmap of proteins associated with MRI activity. Color key represents the relative abundance of proteins that were selected on components 1–2.

Next, we investigated what proteins could discriminate between the three groups of MRI activity by utilizing a PLS-DA. The first two components of PLS-DA included 30 proteins hereof 25 in component one and 5 in component two (Figure 4a). When investigating the functional roles of these proteins, they were associated with inflammatory processes and a majority (57%) of these have been associated with vesicular transport and exosome signaling.

Proteins that tended to be downregulated in the high MRI activity group relative to the low and medium MRI activity groups were protein Z (PROZ) which inhibits blood coagulation; the cytokine TGFβ1 with multiple functions including promotion of Th17 cells in the presence of IL-6 [32]; AHSG that suppresses TGFβ signal transduction [33]; SERPINF1 inhibiting angiogenesis [34]; fetuin B (FETUB) that inactivates the PI3K/AKT signaling pathway that promotes apoptosis and inhibits migration [35]; the extracellular matrix protein-1 (ECM1). Proteins that tended to be upregulated in the low MRI activity group relative to the medium and high MRI activity groups included a number of immunoglobulin proteins; the chemokine CXCL4 (PF4) released by activated platelets promoting coagulation [36]; retinol binding protein 4 (RBP4), which among other functions also increases secretion of proinflammatory cytokines by priming the NLRP3 inflammasome [37]; TTR which interacts with RBP and may also contribute to the regulatory network of coagulation and fibrinolytic balance [38]; the coagulation factor F13B; the complement component C7; thrombospondin-1 (THBS1) interacting with integrins [39] and also activating TGFβ1 [40]; macrophage stimulating 1 (MST1) or macro mammalian sterile 20-like kinase 1; CD5L produced by macrophages with anti-inflammatory function inhibiting TNF and IL-1β production, but also maintaining Th17 cell responses and contributing to pathogenesis of an MS animal model experimental autoimmune encephalomyelitis (EAE) [41].

### 3.2. Significantly Changed Inflammatory and Vascular Markers in Patients with High, Low and Medium MRI Activity

**Figure 5:**
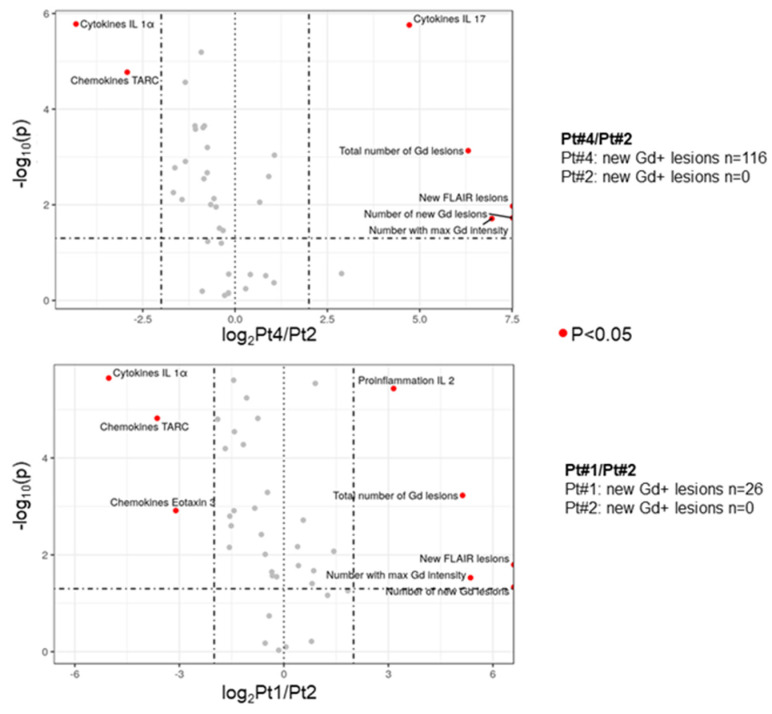
Differences in biomarker levels in MS patients with low and high number of new enhancing lesions. Volcano plot of biomarker expression levels of patient 1 (Pt#1, high number of new enhancing lesions), patient 2 (Pt#2, low number of new enhancing lesions), and patient 4 (Pt#4, medium number of new enhancing lesions). The x axis corresponds to the log2 fold change in biomarker expression of Pt#4 versus Pt#2 (upper panel) and Pt#1 versus Pt#2 (lower panel); Log2 fold changes smaller than −2 and larger than +2 are considered relevant, as indicated by the vertical bars. The y axis corresponds to the negative log10 of the *p*-value of significant difference in biomarker expression for patients 1, 4, and 2; *p*-values smaller than 0.05 are considered significant as indicated by the horizontal bar.

Next, we examined the regulation of inflammatory and vascular markers by differential expression/abundance analysis in patients with high and medium MRI activity. Multiple cytokines were differentially expressed in the plasma of patients with high vs. low MRI activity. IL-17 showed an association with an increased number of lesions and was significantly upregulated at all time points during the 8 weeks in the patient with high MRI activity (Figure 5, upper panel). In the patient with medium vs. low MRI activity, lesions were also significantly increased and there was an elevation of IL-2 at all timepoints during the 8 weeks (Figure 5, lower panel). IL-1α, CCL17/TARC, and CCL26/eotaxin-3 were downregulated in the patients with high and medium activity compared to the patients with low activity (Figure 5).

### 3.3. Weekly Level of Inflammatory and Vascular Markers Related to MRI Activity

**Figure 6:**
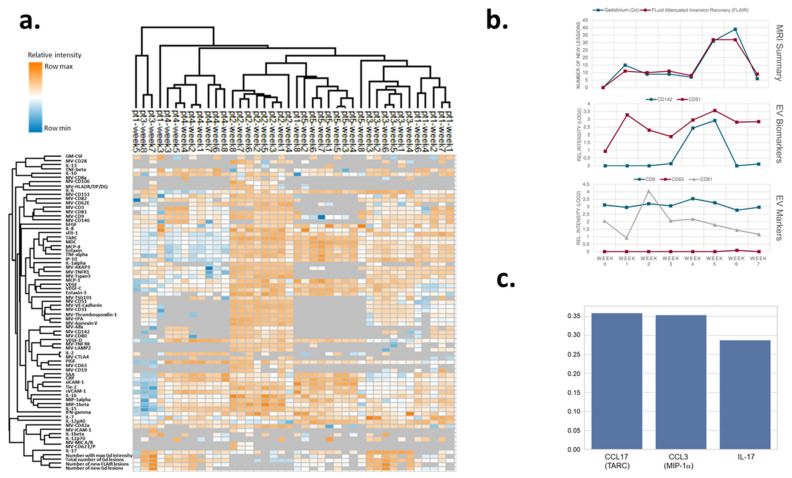
Biomarker levels with MR activity in individual patients and weighted feature importance of the three most important biomarkers. (**a**) Expression levels of soluble pro-inflammatory and EV-associated biomarkers in individual patients at different time points with the highest observed expression indicated by dark orange and the lowest level of a biomarker by dark blue color. Expression levels of each biomarker are standardized across individual patient samples. The dendrograms on top of the chart indicate the clustering structure and similarities of the patient examinations. (**b**) Weekly measurements of MRI-based disease activity of the patients (upper panel) and abundance of new FLAIR and gadolinium-enhancing lesions; (middle panel) corresponding EV-associated protein surface markers (CD142, CD51) measured by the EV array and (lower panel) abundance of EV hallmark surface exosome markers CD9, CD63, and CD81. (**c**) To find the most relevant combination of biomarkers for the prediction of MRI findings, AUC-weighted feature importance was calculated in a cross-validation of the Random Forest model.

We also examined weekly changes in the individual soluble and MRI markers. As expected, all four MRI outcomes clustered together: IL-17 clustered together with the MRI outcomes, and this hierarchical cluster was connected to another close cluster including IL-12p70, IL-1β, and EVs expressing CD62E/P, MIC A/B, ICAM-1, and CD42a (Figure 6a).

Next, we correlated all the soluble multiplex markers with the MRI outcomes (Table 1). The strongest correlation was observed with IL-17: all four MRI outcomes correlated positively with the levels of IL-17 (*p* < 0.001, respectively). A positive association was observed between IL-1β levels and three MRI outcomes (number of new Gd-enhancing lesions: *p* < 0.01; number of new FLAIR lesions: *p* < 0.001; total number of Gd-enhancing lesions: *p* < 0.05). The level of IL-6 positively correlated with the number of new FLAIR lesions (*p* < 0.05). In addition, several chemokines and integrins showed negative associations with the MRI outcomes. The exosome marker TSG101 also positively correlated with the number of new Gd-enhancing and new FLAIR lesions indicating the association of EVs in the plasma with lesion evolution. Indeed, EVs expressing ICAM-1 showed a strong positive association with all four MRI outcomes, although soluble ICAM-1 levels were negatively associated with the same outcomes (Table 2). We also found that an increase in both CD142 and CD51 expressing EVs preceded the evolution of new FLAIR and Gd-enhancing lesions albeit with different dynamics. While CD51-positive EVs were upregulated a few weeks before lesion evolution and remained upregulated, CD142-positive EVs were upregulated prior to the lesion evolution for a short time (Figure 6b). The hallmark EV exosome markers CD9, CD63, and CD81 have similar abundance profiles across the repeated measures.

To find the most relevant combination of biomarkers for the prediction of MRI findings, we calculated weighted feature importance based on Random Forests. The performance of the created models was evaluated using the area under the receiver–operating characteristic curve (AUROC). An ideal, but probably overfitted model, would have an AUROC of 1. The worst outcome would be an AUROC of 0.5, which indicates random classifications. Among the 18 total runs, the AUROC was mostly 1, indicating overfitting, which can be explained by the small sample size. The only condition without overfitting, yet acceptable AUROC seems to be the consideration of only 3 biomarkers for the prediction: IL-17, CCL17/TARC, and CCL3/MIP-1α (Figure 6a). To see whether these three biomarkers are similarly important in the other models, the top four biomarkers for each model were summarized, adding TNF-α to the other three (Table 2). A linear mixed model was also implemented and evaluated. Because of the small sample size only the four most important features (CCL17/TARC, CCL3/MIP-α, IL-17, TNF-α) were considered (Table 3).

## 4. Discussion

In this study, we examined associations of soluble plasma markers with the evolution of new lesions over 8 weeks on frequent, weekly high-resolution MRI with Gd. We were particularly interested in the evolution of inflammatory lesions associated with BBB damage reflected by Gd enhancement. Three methods for inflammation profiling were used to identify markers related to the appearance of new lesions: (i) candidate molecules, such as cytokines, chemokines, and endothelial markers were examined by a multiplex immunoassay; (ii) we used an endothelial-stress optimized EV array to examine plasma exosomes; (iii) quantitative proteomics was applied as a discovery non-targeted approach.

Our first findings by proteomic analysis of the plasma from each timepoint revealed a distinct difference reflecting both disease activity (low, medium, high) but also patient-specific proteome variation changes linked to factors associated with the level of inflammation. Our findings reflect an observed variability between activity states due to the clinical and pathophysiological complexities of MS [42]. None of the patients underwent pharmacological treatment during the time period in focus, hence the acute inflammation remains unaltered. Our data demonstrated a large overlap in both over and under-abundant proteins (Figure 3c) as well as functional biological pathways (Figure 4a–c). Proteins of the innate immune system including acute phase proteins, complement proteins as well as known components associated with neutrophil degranulation such as SAA1, CD14, S100A8, ORM2, and LBP decreased between low-to-high lesion activities whereas regulators of, e.g., platelet degranulation ECM1, FN1, SERPINF2, and HRG increased. Neutrophils are the most abundant circulating and first-responding innate myeloid cells and have so far been underestimated in the context of MS [43]. These innate immune cells are often seen as “first responder cells” during acute inflammation corresponding to our findings comparing protein abundance changes in low-to-high inflammatory activity (Figure 4c). Clinical studies in other diseases indicate that neutrophil extracellular traps (NETs) could act as a novel circulating marker for inflammatory conditions [44]. Using NETs as a new marker requires standardized studies of normal and abnormal levels, which involve measuring cell-free DNA (cfDNA), CitH3, NE, and other NET-related factors in the blood [45,46]. At present, the role and implication of NET activity in BBB disruption remain limited. Recent studies in MS suggest the possibility that activation of NETs would have a cytotoxic effect on the BBB and induce injury of adjacent neurons and other cells of the CNS. Neutrophils have further been detected in the CSF of MS patients at early disease stages and at the beginning of a relapse phase, suggesting their active involvement in the disease [43]. Studies investigating neutrophils isolated from RRMS patients’ blood found these to be more primed, express more inflammatory markers, and display resistance to apoptosis [43,47]. Also, the neutrophil-to-lymphocyte ratio has been proposed as a marker for disease activity, as it was elevated in MS patients and higher in patients experiencing relapse compared to remission [48]. We investigated if the DEPs could discriminate the three MRI activity groups and found several proteins associated with high MRI activity: platelet factor 4 (PF4, CXCL4) is a small cytokine belonging to the CXC chemokine family and apolipoprotein A-I (LPA;apoA1) is the major structural protein component of high-density lipoproteins (HDL) including APO-A4. CXCL4 is among the platelet chemokines including CCL5 (RANTES) and CCL3 (macrophage inflammatory protein-1α) being potent platelet-derived regulators of inflammatory responses [49]. A total of twenty-two proteins were associated with low activity including proteins associated with exosomal and blood microparticle signaling. The medium MRI activity level was differentiated particularly by four proteins (SHGB, HP, SAA1, ECM1) with well-known acute phase and integrin-associated signaling.

Complementary to proteomics profiling, a similar patient grouping was observed by principal component analysis based on 36 soluble markers of inflammation, cell migration, and vascular damage/endothelial activation using multiplex analysis. Plasma markers and proteome profiles clustered the patients and not the MRI activity. Only partial overlap was found among two patients with medium MRI activity, while the third patient clustered separately as did patients with high and low activity. This indicated the individuality of the inflammatory profile of the plasma in the five patients as the new lesions developed, similar to our findings with proteomics.

However, we could observe molecules and pathways that emerged from the analysis of the inflammatory profiling, particularly IL-17 and associated cytokines. One of the molecules we consistently observed in different kinds of analyses was the cytokine IL-17. When we compared the patients with high versus low MRI activity, IL-17 was significantly upregulated at any time point during the 8 weeks. In hierarchical clustering, IL-17 clustered together with the number of new Gd-enhancing and FLAIR lesions, the total number of Gd-enhancing lesions, and the number of lesions with maximum Gd intensity. IL-17 was also one of the markers that positively correlated with all these four MRI outcomes. IL-17 is a signature cytokine of Th17 cells, a CD4+ T cell subset that plays a major role in the initiation of EAE and is considered to be important in the pathogenesis of MS [50]. IL-17 transcript is elevated in chronic silent MS lesions and the IL-17 receptor is also expressed by endothelial cells in multiple sclerosis lesions [51,52] An IL-17-blocking antibody could prevent the development of EAE and suppressed chemokine expression in the brain; EAE was also delayed and attenuated in IL-17-deficient mice [53]. These experimental data indicate the role of Th17 cells in both initiating and maintaining CNS autoimmune inflammation. In MS, acute lesions are characterized by expression of IL-17 mainly related to T cells [51,54]. Stimulated T cells from patients with active MRI lesions produced increased levels of cytokines related to Th17 cells (IL-17, IL-21, IL-22, IL-6, and TNF-α) [55]. The frequency of myelin-reactive peripheral mononuclear cells producing IL-17 was higher in relapsing and especially in untreated patients with secondary progressive MS [56]. The percentage of Th17 cells reflected the clinical activity in MS and they were inhibited by interferon-β treatment, while high IL-17F levels were associated with poor treatment response with clinical or radiological activity [57]. A special subset of T cells, MAIT cells that are present in MS lesions and are preferentially recruited into the CNS during exacerbations produce higher IL-17 in MS compared to healthy controls, and their decline in the peripheral blood coincided with Gd-enhancing lesions [58,59]. Th17 cells migrate more across the BBB than Th1 cells [51]. Natalizumab treatment that decreases the migration of Th1 cells in MS increases the number of Th17 cells and IL-17 levels in the blood. They diminished during rebound upon natalizumab withdrawal and this was accompanied by a parallel increase in IL-17 in the CSF [60]. Fulminant rebound after natalizumab withdrawal can be associated with a high level of blood cells secreting IL-17, and the frequency of such cells correlates with T1-hypointense lesions on the MRI [61]. The monoclonal anti-IL-17A antibody secukinumab significantly reduced the number of cumulative new Gd-enhancing T1 lesions in a randomized clinical trial, while ustekinumab inhibiting both IL-12 and IL-23 had no such effect [62]. The frequency of Th17 cells was increased in the CSF during relapse [63,64]. Serum IL-17 was also different between MS and controls in a recent meta-analysis [65].

One of the major pathological effects of Th17 cells in EAE and MS is the disruption of the BBB [51]. Th17 cells produce cytolytic enzymes such as granzyme B that promote BBB disruption and recruitment of other lymphocytes [51]. Increased levels of IL-17A in the CSF correlated with albumin quotient, a marker of BBB integrity, and rhIL-17A in the presence of IL-6-impaired endothelial cell monolayer integrity [64]. IL-6 is a major cytokine contributing to BBB disruption and is also necessary for the pathogenic differentiation of Th17 cells [66,67]. A special subset of monocytes migrates across the inflamed BBB, specializes into dendritic cells, is present in acute MS lesions, and promotes Th17 cells in the lesions by producing IL-6 [51]. Both IL-6 and its receptor are expressed in MS lesions, and CSF levels decreased after treatment switch to rituximab in MS patients [68,69]. Indeed, we also found that the level of IL-6 correlated with the number of new FLAIR lesions in our study.

In addition to IL-17 and associated IL-6, we also found IL-1β correlating with the development of new and enhancing lesions. Th17 cells can induce IL-1β production in macrophages and dendritic cells, can also contribute to BBB disruption, and are present in the CSF of MS patients [70,71]. Chronic expression of IL-1β in the CNS induced recruitment of neutrophils, breakdown of the BBB, activation of microglia and astrocytes, and extensive demyelination [72]. IL-1β is upregulated in MS lesions [68,73,74]. CSF level of IL-1β correlated with cortical and WM lesion load but not with the presence of Gd-enhancing lesions in a cross-sectional study [75]. CSF IL-1β levels in remission did not predict new MRI lesion formation but were associated with disease progression in a longitudinal study [76].

When addressing possible composites for the prediction of new lesion evolution by different models, the most important features seemed to be, again, IL-17 in addition to CCL17/TARC, CCL3/MIP-1α, and TNF-α. From these models, around 24.5% of the variance in the number of new FLAIR lesions is explained by the four biomarkers. Interestingly, while this composite of four biomarkers predicted new lesion evolution, only IL-17 was positively associated with lesion appearance, the other three biomarkers showed a negative association. Soluble TNF-α can activate TNF1 receptors and exert proinflammatory effects, but soluble TNF-α receptors may interfere with such effects [77]. In this study, we did not measure soluble TNF receptors. Our data also emphasize that composite biomarkers may have very different predicting potentials compared to individual biomarkers within the composite, as we have shown previously in a proteomics study [78].

While the IL-17 pathway emerged in our study as a major pathway associated with the development of new and enhancing lesions, cytokines related to Th1 cells were not associated with the MRI outcomes. However, chemokines CCL3/MIP-1α and CCL4/MIP-1β, which may reflect Th1 activation, negatively correlated with all four MRI outcomes, while CXCL10/IP-10 did not. Unexpectedly, in our study evolution of new and Gd-enhancing lesions was negatively associated with the plasma level of integrins, adhesions molecules, and chemokines. Of note, an inverse direction of association was observed with the MRI outcomes of soluble ICAM-1 and the endothelial EVs expressing ICAM-1: while soluble ICAM-1 showed a strong negative correlation, ICAM-1^+^ EVs showed a strong positive correlation with lesion evolution. It is possible that membrane-bound ICAM-1 on endothelial cells reflected by the shedding of ICAM-1^+^ EVs may indicate more endothelial activation/stress related to lesion evolution in the acute phase than secreted soluble ICAM-1 which may have heterogeneous cellular source [79].

Besides soluble markers, we investigated exosome-associated protein surface markers by an optimized EV array sensitive to EV hallmark surface markers, immune system specific as well as specific markers for endothelial cell stress and oxidative stress markers [12]. A significant contribution to the immunoregulatory events by BBB disruption and neuroinflammation may derive from a cell-to-cell communication system involving the production, secretion, and transfer of extracellular vesicles known as exosomes [80]. The array consistently quantitatively measured 33 surface-exposed protein markers in multiple functional groups for phenotyping the patients from baseline to day 49 using the EV hallmarks CD9, CD63, and CD81 [12]. CD142 and to a lesser degree CD51 correlated to the weekly FLAIR abundance in the patients, however, slightly earlier than the MRI-based activity indicating potential impact as an early marker of disease activity (Figure 6b). Thromboplastin, or CD142 is a 45 kD type I transmembrane glycoprotein and an important “extravascular” tissue factor (TF) where induction of soluble TF is stimulated during various chemokine- and cytokine-induced inflammatory states [81]. It is expressed on the surface of a variety of cells that are physically separated from the circulating blood. The breakdown of BBB that characterizes the MS disease process exposes the TF of astrocytes, which can promote activation of the coagulation cascade. The cascade, in turn, requires activated membranes to support biochemical reactions, canonically provided by platelets and also supported by platelet abundance changes in our proteomic data. CD51, also called integrin alpha ν, is a heterodimeric integral membrane protein that also correlates with fluctuations of new lesions. This integrin plays a role in diverse biological processes such as cell migration, tumor invasion, bone resorption, angiogenesis, and immune responsiveness. Endothelial cells release microparticles <∼1.5 μm (EMP) during activation or apoptosis. Previous studies identified a CD31 + EMP positive association with gadolinium enhancement in patients with MS in remission whereas CD51+ EMP remained elevated in both exacerbation and remission [82]. This study suggested that CD31 + EMP is a marker of acute injury, whereas CD51 + EMP reflects chronic injury of the endothelium. This supports our finding of correlating levels of CD51+ exosomes and the degree of new lesion activity.

One limitation and weakness of our study is the absence of matched healthy controls, so we could not establish if levels of serology markers associated with MRI outcomes of these healthy subjects were increased in the plasma. This study aims to further investigate extensive MRI profiling and serology findings with detailed MRI findings in the early acute phase of these patients. Although the number of patients is low, the collected number of samples (*n* = 40) is reasonable for the detailed examination of BBB disruption and lesion evolution in a highly unique setting. Similarly, the number and gender distribution of the cohort remains limited. In addition, CSF samples were not examined in these five patients at the time of inclusion.

## 5. Conclusions

In conclusion, we used a unique dataset of weekly MRI with gadolinium combined with proteomics, multiplex assay of soluble inflammatory and EV markers in serial plasma samples to address the relationship between systemic inflammatory changes and acute lesion evolution in the brain. The proteomic profiling of plasma samples revealed a low intra-variability of the individual patients indicative of large patient-to-patient variability. We found that altered key processes were associated with acute adaptive and innate inflammatory responses, neutrophil degranulation, complement, and coagulation pathways. Several analyses of our dataset indicated the association of systemic IL-17 with the evolution of new Gd-enhancing and FLAIR lesions on MRI suggesting that this cytokine and the Th17 pathway may be important in BBB disruption and initiating inflammation in the brain of patients with MS. 

## Figures and Tables

**Table 1:** Association of soluble and EV marker levels with lesion evolution on the MRI.

Marker	Correlation with the number of			
	New Gd^+^ Lesions	New FLAIR Lesions	Total Gd^+^ Lesions	Lesions with Maximum Gd^+^
Tie 2	−0.56079 ***	−0.59616 ***	−0.74088 ***	−0.63491 ***
VEGF-C			0.35236 *	−0.36838 *
VEGF-D	−0.38390 *	−0.42184 *	−0.36100 *	−0.34755 *
sFLT-1			−0.46322 **	−0.42402 **
CCL11, eotaxin			−0.41500 **	−0.31424 *
CCL2, MCP-1			−0.32893 *	
CCL13, MCP-4			−0.44751 **	−0.36185 *
CCL22, MDC			−0.43846 **	−0.33729 *
CCL3, MIP-1a	−0.39215 *	−0.42601 *	−0.54656 ***	−0.40649 **
CCL4, MIP-1b	−0.34290 *	−0.37318 *	−0.48321 **	−0.40504 **
CCL17, TARC	−0.38653 *	−0.40675 *	−0.55210 **	−0.39932 *
sICAM-1	−0.36958 *	−0.40194 *	−0.49975 **	−0.39018 *
sVCAM-1		−0.35616 *	−0.36749 *	−0.35089 *
IL-15	−0.37681 *	−0.40334 *	−0.46042 **	−0.46021 **
IL-16	−0.35487 *	−0.36831 *	−0.48427 **	−0.39520 *
TNF-a	−0.36675 *	−0.38338 *	−0.59531 ***	−0.49169 **
**IL-17**	0.64576 ***	0.71139 ***	0.68207 ***	0.47769 **
**IL-1b**	0.47589 **	0.52346 ***	0.31339 *	
**IL-6**		0.35851 *		
EV-LAMP2			−0.40376 **	
**EV-TSG101**	0.34669 *	0.35821 *		
**EV-ICAM1**	0.65587 ***	0.57176 ***	0.57701 ***	0.73735 ***

* *p* < 0.05; ** *p* < 0.01; *** *p* < 0.001; Positive correlations are shown in bold.

**Table 2:** The importance of the 4 biomarkers in a composite to predict new lesion evolution.

Biomarker	Most important ^a^	2nd Most Important ^a^	3rd Most Important ^a^
CCL17 (TARC)	**10**	5	3
CCL3 (MIP-1a)	6	**8**	4
IL-17	2	5	**7**
TNF-a	0	0	4

^a^ To find most relevant combination of biomarkers for the prediction of MRI findings, weighted feature importance based on Random Forests was calculated. The importance of the top 4 biomarkers for each model are shown.

**Table 3:** Performance of regression models to predict lesion evolution by a composite of 4 biomarkers: CCL17 (TARC), CCL3 (MIP-1α), IL-17, TNF-α.

Model (Number of Lesions Types)	R-Squared ^a^	RMSE (MSE)
New Gd+ lesions	0.015	3.585
New FLAIR lesions	0.245	2.868
Total Gd+ lesions	0.0197	21.478
Lesions with max Gd intensity	0.085	11.511

^a^ A linear mixed model was implemented and evaluated to predict a value for the number of new lesion types while taking effects of patients and timepoints into account. The four most important features from 4 biomarkers (CCL17/TARC, MIP-α, IL-17, TNF-α) were considered (see Table 2 and Figure 6). RMSE=root mean squared error

## Data Availability

All raw data and unfiltered search data have been made public through the PRIDE ProteomeXchange consortium with the dataset identifier PXD035422. Other data is available by personal request to the corresponding authors.

## Supplementary Materials

The following supporting information can be downloaded at: https://www.mdpi.com/article/10.3390/biomedicines11123170/s1, The MS proteomics data have been deposited to the ProteomeXchange Consortium via the PRIDE partner repository with the dataset identifier PXD035422.
